# Community dynamics can modify the direction of simulated warming effects on crop yield

**DOI:** 10.1371/journal.pone.0207796

**Published:** 2018-11-19

**Authors:** Mark A. K. Gillespie, Marco Jacometti, Jason M. Tylianakis, Steve D. Wratten

**Affiliations:** 1 Department of Engineering and Natural Science, Western Norway University of Applied Sciences, Sogndal, Norway; 2 Bio-Protection Research Centre, Lincoln University, Lincoln, New Zealand; 3 Bio-Protection Research Centre, School of Biological Sciences, University of Canterbury, Christchurch, New Zealand; 4 Department of Life Sciences, Imperial College London, Silwood Park Campus, Ascot, Berkshire, United Kingdom; Universidade Federal de Uberlândia, BRAZIL

## Abstract

Climate change affects agriculture through a range of direct and indirect pathways. These include direct changes to impacts of pests and diseases on crops and indirect effects produced by interactions between organisms. It remains unclear whether the net effects of these biotic influences will be beneficial or detrimental to crop yield because few studies consider multiple interactions within communities and the net effects of these on community structure and yield. In this study, we created two experimental grapevine communities in field cages, and quantified direct and indirect effects of key pest and disease species under simulated climate change conditions (elevated temperature and reduced humidity). We found that the net impact of simulated climate change on total yield differed for the two communities, with increased yield in one community and no effect in the other. These effects, and the interactions between pests and pathogens, may also have been affected by the prevailing abiotic conditions, and we discuss how these may contribute to our findings. These results demonstrate that future research should consider more of the interactions between key organisms affecting crops under varying abiotic conditions to help generate future recommendations for adapting to the effects of climate change.

## Introduction

Climate change affects agriculture through a range of direct and indirect pathways [1, 2]. These include direct changes in impacts of pests and diseases on crops [3], which may be mitigated indirectly by altered attack rates by natural enemies of the pest and insect vectors of fungal pathogens [4]. However, it remains unclear whether the net effects of these biotic influences will be beneficial or detrimental to crop yield [1, 5]. In this study, we aimed to address this lack of knowledge by generating two experimental grapevine communities in field cages, and quantifying the combined effects of key pest and disease species under simulated climate change conditions (elevated temperature and reduced humidity).

Recent and projected climate conditions create a constant challenge to agriculture and food security [1]. However, for some crops at certain latitudes, increased mean temperatures may improve yield, while climate change may lead to crop heat stress and water shortages in areas where air temperatures are already close to crop maxima [1]. Furthermore, crops may face greater pressures from antagonists under future climate regimes [3]. With increasing temperatures, many invertebrate pests are able to expand their geographical ranges, shift the timing of their emergence to more vulnerable crop growth phases and increase their development rates and number of generations per year [5]. Plant pathogens are also able to take advantage of changing conditions, with increased fecundity resulting from elevated temperature, and the spread and phenology of diseases also likely to be altered [6].

Much is known about the probable impact of certain climate change drivers on individual species or pairwise species interactions [2, 4, 7]. However, there is uncertainty regarding how these effects interact, and whether species’ indirect effects on plants may contrast with their direct effects. This makes insights from studies of single species insufficient for making realistic projections of future food production. Besides antagonist crop-pathogen or crop-pest interactions, other mutualistic, facilitative or competitive interactions are found in agricultural ecosystems, and recent theory suggests that merging these into experimental approaches can give more realistic insights into community processes [8]. For example, herbivores can also serve as vectors of plant pathogens [2, 5] and may benefit from pathogen infection of the plant [9]. It is not known how such associations will affect, or be indirectly affected by, the response of natural enemies of herbivores to climate, but previous findings that plant pathogens can structure insect communities [10] suggest that such complex effect pathways may be important.

To meet the challenges of understanding the influence of future climate conditions on agriculture, research urgently needs to address the interplay between multiple abiotic and biotic stresses on crop yield [11]. In this study, we assess the effects of simulated climate change on two experimentally-assembled food webs in grapevines (*Vitis vinifera* (L.)) in New Zealand. Our model crop is chosen for its economic importance, reliance on favourable abiotic conditions and consequent sensitivity to climate change. Elevated temperatures can lead to enhanced flowering in grapevines [12], but reduced water availability increases plant stress, outweighing the positive impact on yield [13, 14]. Such stress can also make grapevines more vulnerable to attack from pests and disease through reduced resistance [15]. However, the extent to which these different influences interact with one another remains unclear.

The two experimental communities used in this study contained important pests that may also act as pathogen vectors. As a result, the communities include varying levels of naturally occurring key pathogens of grapevines. Previous work has demonstrated effects of climate change drivers on these species individually or on pairwise interactions (see Study system section below). Importantly though, no previous studies appear to have considered the impact of real or simulated climate change on the multiple interactions between crops, pests and pathogens in agricultural food webs. To address this, our research aimed to answer the following questions:

1. What are the impacts on grape yield of simulated temperature increase and reduced humidity, and of the introduced insect treatments?
2. What are the impacts of these treatments on the cover of important pathogens of grapevines?
3. How do simulated temperature increase and reduced humidity, introduced pests and naturally occurring pathogens combine to impact crop yield and the percentage of clean (unaffected by pathogens) grape bunches?

The first question is addressed with simple mixed model analyses, and we particularly expect to see a negative impact of the climate treatment on crop yield due to plant water stress. Secondly, we expect that pathogens will also respond positively to enhanced temperatures and the presence of their insect vectors. The third question is explored using path analysis to disentangle the individual and interactive responses of the members of each food web. This analysis provides a means to estimate the magnitude and significance of hypothesized causal links and indirect pathways between variables [16]. Using this method we can demonstrate how climate may affect crop yield through a range of pathways, and identify the factors that need to be considered together to arrive at estimated impacts of climate change on food production.

## Materials and methods

### Study system

We used two initial communities to ensure that our findings were not specific to a single system. The focal organisms of these study systems are key functionally- and economically-important species common in many vineyard regions worldwide, and are likely to be affected by climate change. In the first experiment, we introduced larvae of the light brown apple moth (*Epiphyas postvittana* (Walker) (Lepidoptera: Tortricidae), hereafter referred to as “LBAM”), a major horticultural pest that feeds on nearly all types of fruit crops, ornamentals, vegetables and glasshouse crops [17]. In addition, we introduced adults of two natural enemies of LBAM to certain cages (see below): 1) *Dolichogenidea tasmanica* (Cameron) (Hymenoptera: Braconidae), the predominant and most effective parasitoid of the moth [18], and 2) European earwig (*Forficula auricularia*) (Linn.) (Dermaptera: Forficulidae), a key predator of LBAM (as well as other pests), removing as many LBAM larvae as all other predators combined [19]. In the second experiment, we introduced into certain cages the citrophilus mealybug *Pseudococcus calceolariae* (Maskell) (Hemiptera: Pseudococcidae), a small phloem-feeding insect common among grapevines in New Zealand vineyards [20] and a vector of closteroviruses associated with grapevine leafroll disease [21]. In this experiment we also included the southern ant, *Monomorium antarcticum* (Smith) (Hymenoptera: Formicidae), which has a mutualistic association with mealybugs, harvesting the honeydew that the pest excretes in exchange for protection from natural enemies [22–24]. As poikilotherms, the insect species studied here were expected to respond positively to temperature changes [11]. For example, the LBAM appears to have an upper temperature tolerance limit which is within the range of predicted climate change in warmer regions [25]. Similarly, although we lack species-specific information on temperature tolerance of the mealybug species studied here, work on a similar species has revealed positive effects of temperature on development and density [5].

In both food webs, a number of pathogens also occurred naturally and we collected data on their abundance. These included powdery mildew (*Uncinula necator* (Burill)), a major disease of grapevines affecting both leaves and fruit, which increases its rate of development in response to elevated temperature and low rainfall [26]. Previous work has shown that climate change simulation can positively affect this pathogen [27], although neutral effects have also been found [26]. *Botrytis cinerea* (Pers.) was also present in both food webs, and is a widespread disease that infects grapevines and has greater impacts in moist environments at relatively low temperatures [28, 29]. Powdery mildew is not known for its interaction with invertebrates on grapevines (although see [30]), but we expected interactions between *Botrytis* and insect pests in both years. In addition to the facilitation of this disease by the LBAM, which can act as a vector [9], the presence of ants and mealybugs (in the second food web) can increase *Botrytis* infection. Mealybug infestations can also facilitate the spread of sooty molds, which were present in the second food web [28].

### Site description (both years)

The experiment was conducted in a research vineyard at Lincoln University, Canterbury, New Zealand (43°38 S, 172°27 E, 9 m ASL). The grapes were unirrigated, 16 year old Chardonnay Mendoza, on their own rootstocks, managed to commercial standards, and spur-pruned and head trained to achieve a “goblet” vine training system independent of trellis [15] (See S1 Appendix for full details). This system was adopted so that the trellis could be removed to allow the field cages to be placed over the vines without impediment.

### Experimental design

Our two selected webs were studied in consecutive field seasons using field cages. Each cage was constructed of a metal frame with a basal area of 1.8 x 1.8 m and a height of 2.0 m. The cages were covered with a net (mesh size: 0.28 x 0.78 mm) and erected and secured over experimental vines approximately 2 m apart. To simulate climate change conditions, half of the cages being used in each year had a transparent polyethylene cover placed over the top, also covering the upper 1 m of the cage sides. Humidity and temperature data were measured in each cage every 15 minutes with Hygrochron iButton DS1923 dataloggers (maxim integrated). The covers reduced heat convection and prevented direct rainfall and therefore reduced cage relative humidity and increased temperature (Table 1), producing conditions predicted by climate change models for the study region [31]. For example, in a rapidly decarbonizing scenario, New Zealand temperatures are expected to increase by 0.73°C by 2030–2049 and by 1.10°C by 2080–2099 (model average predictions).

**Table 1:** Mean and standard error of the temperature and humidity in cages with and without plastic covers in each year, with confidence intervals (CI) and results of a Welch’s t-test for the differences (degrees of freedom modified due to unequal variances).

			N	Mean	Standard Error	Difference	Confidence Intervals	t	d.f.	p
**Year 1**	**Temperature (°C)**	Plastic	18	17.3	0.08	0.8	0.65; 1.10	8.01	34	<0.001
		No plastic	18	16.5	0.08					
	**Humidity (%)**	Plastic	18	73.5	0.26	-1.7	-2.34; -1.16	-6.16	24.1	<0.001
		No plastic	18	75.2	0.12					
**Year 2**	**Temperature (°C)**	Plastic	15*	13.8	0.16	1.1	0.76; 1.48	6.53	18.3	<0.001
		No plastic	16	12.7	0.06					
	**Humidity (%)**	Plastic	18	79.9	0.59	-3.5	-4.78; -2.20	-5.73	16.1	<0.001
		No plastic	18	83.4	0.16					

*one data logger failed to record

### Year 1 experiment

In September 2009, the first field season was set-up with 36 cages erected over individual vines. The vines within the cages were initially treated with the insecticide dichlorvos (Nuvos, 0.175% 2,2-dichlorovinyl dimethyl phosphate, 1–1.5L per cage) to kill all resident arthropods and left for six days. Dichlorvos was chosen for this purpose because of its broad-spectrum activity [32] and short persistence in the environment (95% reduction in concentration in the first 20 minutes [33]). All cages were arranged in a 3 block/replicate factorial, randomized block design, with twelve treatment combinations comprising the presence or absence of a) plastic covers, b) light brown apple moth, c) the European earwig (*F*. *auricularia*) and d) the parasitoid *D*. *tasmanica* (Table 2). There were 36 observations in total for this experiment.

**Table 2:** Treatment combinations for the first experiment. The design was not a full factorial experiment, with omitted treatments being those including the parasitoid (*Dolichogenidea tasmanica*) as the only insect, and both the earwig (*Forficula auricularia*) and parasitoid without the LBAM (*Epiphyas postvittana)*. Shaded cells = presence of treatment, unshaded cells = absence of treatment. The treatments were repeated 3 times (total number of cages = 36).

Treatment Number	Plastic covers	LBAM larvae	Earwig adults	Parasitoid adults
1				
2				
3				
4				
5				
6				
7				
8				
9				
10				
11				
12				

On December 21 2009, four batches each of between 55 and 65 LBAM eggs laid on paper (purchased from Plant and Food Research Limited, Mt Albert, New Zealand) were stapled to the underside of each of four vine leaves, approximately 1m from the ground and equally spaced around the perimeter of each of the vines in the LBAM-treatment cages. On January 1 2010, four pairs (male and female) of adult *D*. *tasmanica* aged between 4 and 12 days (from laboratory cultures; see S1 Appendix), and four pairs of earwigs of equal weight (collected from an orchard at Lincoln University; see S1 Appendix), were added to the appropriate cages. Any arthropods, other than those species introduced to the cages, that had entered the cages since the dichlorvos spraying were recorded and then killed by hand on a weekly basis. On February 8 2010, just before the beginning of the experiments, the vines were sprayed with Systhane 400 WP with a knapsack sprayer to control powdery mildew. This was done so that incipient mildew infections were reduced uniformly to very low levels at the start of the experiment.

On April 9 2010, a sample of ten grape bunches, of the same variety and history as the vines used in this experiment, was removed from adjacent vines and assessed for sugar concentrations with an optical refractometer to assess if the grapes were ready to be harvested. At this time, the mean sugar concentration was 20.7° Brix and considered ready for harvest. Over the following week, soil moisture and plant health were measured in each cage. Soil moisture was measured with an electronic soil moisture meter (Campbell Scientific “Hydrosense” hand held display CD620 and CS620 12 cm probe) in four locations per cage, each approximately 30 cm from the vine trunk and equally spaced around the vine. The measurements were made between 10.00–11.30 h to allow dew to have evaporated. Plant health (leaf chlorophyll content) was measured by with a SPAD (Soil Plant Analysis Development) meter (Konica Minolta SPAD-502 (Osaka, Japan)) on twenty young leaves per vine from the top and sides of the vines’ outer foliage. SPAD was used as it is a good indicator of water stress [15, 34] and temperature stress [35, 36].

Subsequently, the grape bunches were removed from the vines, and final values of remaining variables were established. Grape bunches were counted and weighed to provide two estimates of total yield, and they were examined to count the number of LBAM larvae, estimate the amount of LBAM larvae damage to berries, and to estimate the percentage of bunches infected with *Botrytis* bunch rot and/or powdery mildew infection. Three days later, all the leaves were removed from the vines and each leaf was assessed for the presence of LBAM larvae and *D*. *tasmanica*. Final earwig numbers were counted and recorded in each cage using corrugated cardboard rolls that had been placed around the trunks of the vines following the methodology used in [37].

### Experimental design: Year 2

The second field season’s experiment was set up in the same way except for the following details. In September 2010, 32 of the same cages described above were erected over different individual grape vines from those used in the previous year, but of the same variety and from the same vineyard block. All cages were arranged in a full-factorial, randomized block design, with eight treatments comprising the presence or absence of a) plastic covers, b) ants and c) mealybugs. Thus there were 32 observations for this experiment, with four replicates of each treatment. On January 19 2010, a potato slice (cv. Désirée) with approximately 60 mealybugs, of mixed age and sex, was attached with a cable tie to a single grape bunch in the middle of the canopy on the northern (warmer) side of each vine in the mealybug treatment. The mealybugs came from a laboratory culture founded with individuals purchased from Zonda Resources Limited, Pukekohe, New Zealand (see S1 Appendix). This bunch was then covered for one week with a 15 × 30 cm cotton bag, with a mesh size of 0.3 × 0.3 mm, open at one end and tied with cable ties. This allowed the mealybugs to move from the dehydrating potato slice into the bunches and establish without the threat of predation or their moving to other parts of the plant or falling to the ground before selecting a vine feeding site. From January 26–28 2011, ant colonies were introduced into the appropriate cages, in one block per day over four successive days. Colonies were collected from under concrete paving in a local domestic garden. Full details of organism origins can be found in the S1 Appendix.

On April 22 2011, following the protocol used in the previous year, bunches from grape vines adjacent to the field cages were tested for sugar concentration and considered ready for harvest when a mean of 22.4° Brix was reached. Over the following week, leaf chlorophyll content, vine yield, *Botrytis* bunch rot and powdery mildew infection were measured according to the protocol followed in the previous year. Soil moisture was not measured in year 2 because of financial and logistical constraints. The percentage of bunches infected with sooty mold infection was assessed using the same protocol as for the other plant pathogens. Mealybug populations were assessed by counting individuals in bunches. Ant colonies were excavated then separated into colonized and non-colonized soil. The volume of the colonized soil was then measured in 300 mL beakers. This was done on a cool morning, as at this time the colony was less active and most of the workers would still have been within the colony.

### Data analysis (both years)

Variables that did not appear to fit a normal distribution following a visual inspection, were log_10_, logit or square root transformed as appropriate (i.e., only proportion data were logit transformed). The data were then analyzed using Linear Mixed Effects models using the *nlme* [38] package in the R programming environment (version 3.3.3; [39]). Models were initially created to test the first research question concerning the effect of the treatments on three measures of grape yield: 1) total yield, 2) number of bunches and 3) percentage of clean bunches (those not affected by pathogens or LBAM larvae damage). Therefore, three models were constructed for each year, with block as the random effect and the treatments as categorical independent variables. Interactions between treatments were not included due to the small sample sizes and a lack of evidence of such effects when explored graphically. In some models, the residuals suffered from heteroscedasticity and where we could identify structure in these patterns, we added a variance structure function (varIdent function) to allow variances to differ between levels of a factor [40]. This issue affected the three Year 1 models, and allowing variances to differ between the levels of the *D*. *tasmanica* treatment greatly improved the residual patterns. All models were simplified to minimum adequate models through backwards stepwise selection and compared using AIC. The exception to this was the model for percentage of clean bunches in Year 2. In this year, there were very few clean bunches resulting in a high number of zeros for this variable. It was therefore converted to a binary presence/absence response variable and a generalized linear mixed model was fitted with binomial error distribution using the glmer function of the *lme4* package [41].

In addition, a series of models were constructed for each year, again with the yield variables as response, to test the impact of other pressures present in the cages. In year one, the explanatory variables were *Botrytis* bunch rot cover, powdery mildew cover, number of LBAM larvae and plant health (SPAD). Soil Moisture was also used in place of plant health, as these two variables were highly correlated and it was not possible to include them in the same model. In year two, the explanatory variables were *Botrytis* bunch rot cover, powdery mildew cover, number of mealybugs, sooty mold cover and plant health (SPAD). Final minimum adequate models were selected in the same way as above.

To answer the second research question, a third series of models were constructed to test whether the abundance of pests and pathogens at the end of the experiment were affected by the experimental treatments. In these models, the pest or pathogen variables were response variables, the treatments were explanatory variables, block was random effect and the model selection was completed as in the first set of models. Heteroscedasticity in the residuals was again addressed by adding a variance structure where appropriate (Year 1, powdery mildew model and Year 2, botrytis model). There was one exception again: the sooty mold variable had a high number of zeros, and this was converted to a binary presence/absence variable and analysed with a generalized linear mixed model with binomial error distribution. This model had convergence problems, so the mealybug treatment was represented by final mealybug numbers rather than presence/absence.

**Fig 1:**
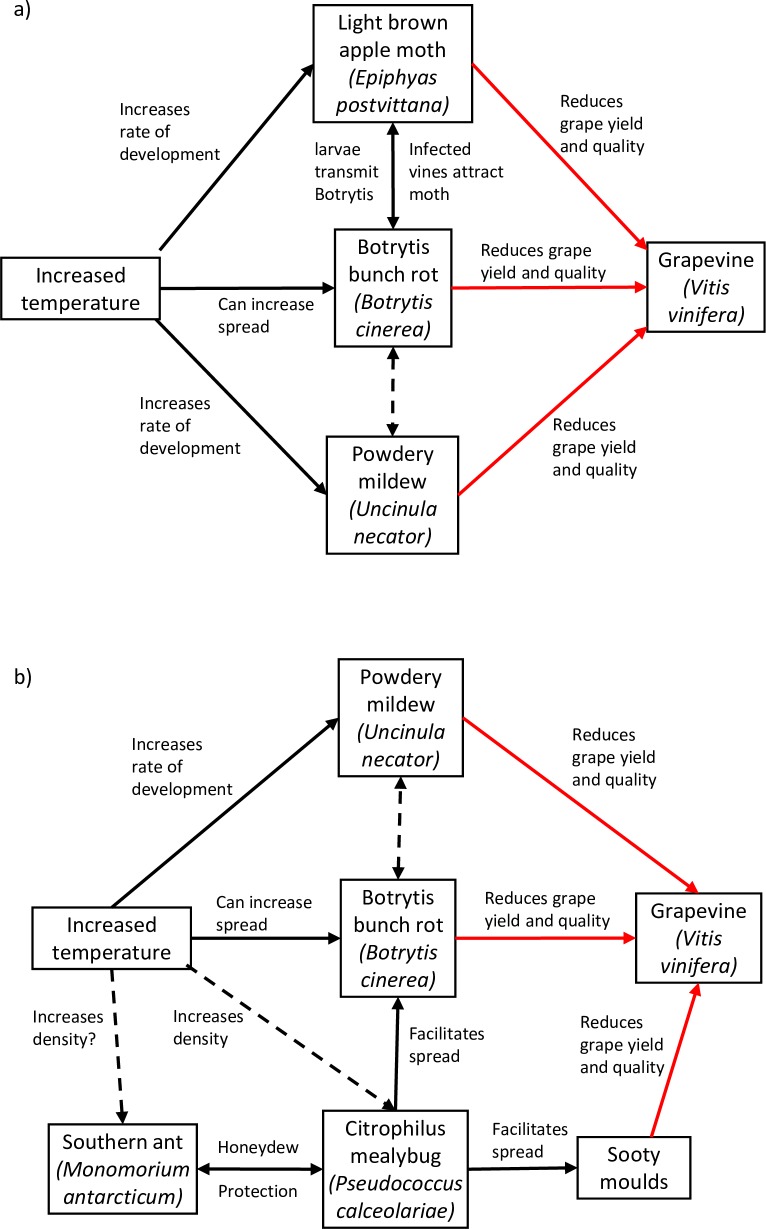
**Diagram of the food webs studied during the two field seasons**, 2009/2010 (a) and 2010/2011 (b). The direction of single headed arrows indicates likely pest or disease pressures; double headed arrows indicate possible or known mutualistic relationships; dashed arrow indicates hypothesised relationship and solid arrows are known relationships from the literature.

Following the results of the mixed models, we hypothesized that the simulated climate treatment may impact the response variables through indirect effects. We therefore used path analysis to explore direct and indirect relationships among variables. It should be noted that we use path analysis here as an exploratory tool, rather than as a confirmatory analysis technique. To do this we used the *piecewiseSEM* [42] and *nlme* [38] packages in the R programming environment (version 3.3.3). The piecewise SEM (structural equation modelling) approach to path analysis allows for hierarchical experimental designs with smaller data sets, because the path diagram is represented by a series of linear mixed effect models, evaluated individually rather than simultaneously as in covariance-based SEM [16, 42]. For each experiment, we constructed three path models, with total yield as the focal yield variable. The first included paths between variables only where our initial analyses above identified significant effects. The “basis set” of linear equations for these path models (the minimum set of conditional independence claims) were therefore solved as the linear mixed effects models described above, using Block as the random factor. In these models the increased temperature/reduced humidity treatment was converted to a dummy variable (i.e. 1 = presence of treatment, 0 = absence). The *psem* function of the *piecewiseSEM* [42] R package conducts d-separation tests [43], which essentially test for missing paths between the variables. Where missing paths were suggested, they were added to the basis set for the second path models. The third set of models we considered included additional pathways that we expected to be important in the system using logic and evidence in the literature. These hypothesized pathways are depicted in Fig 1 and outlined in the *Study system* section above. Model fit between the three models for each experiment was evaluated using Fisher’s C, a measure of whether the model is a good fit to the data, and AICc, the Akaike Information Criterion corrected for small sample sizes. The three models were also repeated for percentage of clean berries as the yield variable of focus. However, due to the large number of zeros for this variable in Year 2, we were unable to construct a satisfactory path model, and the results are not presented. The results of the final models are presented in Tables B—K in S1 Appendix.

## Results

### Year 1

The linear mixed effect models with the three yield response variables and treatments (presence or absence of plastic covers, LBAM, *D*. *tasmanica*, earwigs) showed few significant effects of the treatments on yield (Table 3). There were weak effects of the *D*. *tasmanica* treatment on total yield and number of bunches, where the presence of the parasitoid appeared to have a positive effect on both response variables (Table 4). However, these results should be interpreted with caution because the significance is low, this factor was unbalanced in the experimental design (present: n = 7, absent: n = 29) and the variances for the two levels of the factor are unequal (although the models have corrected for this). There was also a weak significant effect of the temperature treatment on the number of bunches with increased temperature and reduced humidity having a positive effect (Table 4). In the second series of models testing the impact of other pressures on yield, there was a significant positive effect of plant health on the percentage of clean bunches (Table 4). Plant health (SPAD) has a strong positive correlation with Soil moisture (Spearman’s ρ = 0.854, p < 0.001), and a linear mixed effect model with the latter as independent variable resulted in a similar significant positive effect (results not shown).

**Table 3:** Analysis of variance tables. Anova tables using Type II sums of squares for Linear mixed effects models of three yield response variables against experimental treatments. Explanatory variables listed are presence/absence factors.

	Year 1			Year 2		
Response	Explanatory	Y^2^	p	Explanatory	Y^2^	p
Yield (log)						
	Temperature/ humidity treatment	0.09	0.76	Temperature/ humidity treatment	20.16	**<0.001**
	LBAM larvae	0.39	0.53	Mealybugs	1.59	0.21
	*D*. *tasmanica*	26.30	**0.01**	Ants	2.44	0.12
	Earwigs	0.97	0.33			
Percentage Clean Bunches						
	Temperature/ humidity treatment	0.41	0.52	*Temperature/ humidity treatment	1.08	0.30
	LBAM larvae	0.59	0.44	Mealybugs	0.15	0.70
	*D*. *tasmanica*	0.43	0.51	Ants	0.00	0.96
	Earwigs	1.11	0.29			
No Bunches (sqrt)						
	Temperature/ humidity treatment	4.83	**0.03**	Temperature/ humidity treatment	1.28	0.26
	LBAM larvae	1.65	0.19	Mealybugs	0.00	0.98
	*D*. *tasmanica*	6.25	**0.01**	Ants	0.12	0.73
	Earwigs	0.46	0.50			

*Percentage clean bunches was converted to presence/absence in Year 2 due to the high number of 0 values, and these results are from a binomial generalised mixed model.

**Table 4:** Results from mixed effects models, Year 1. Model results for the three yield variables in Year 1 against treatments or Plant Health (Percentage of Clean Bunches only). These are the minimum adequate models following backward stepwise selection of full models including all treatments (Yield and Bunches) or all pathogen, pest and plant health variables (Percentage of Clean Bunches) as explanatory variables. The remaining main effects are those that had a significant effect on the response. Other models described in the text are not presented due to non-significant effects.

Response		Estimate	s.e.	t	p
Percentage of Clean Bunches	Intercept	-12.07	8.36	-1.44	0.16
	SPAD	1.06	0.37	2.87	**0.007**
Yield (log)	Intercept (absent)	2.53	0.08	30.91	**<0.001**
	*D*. *tasmanica* (present)	0.27	0.09	3.03	**0.005**
No. bunches (square root)	Intercept (both absent)	4.54	0.32	14.15	**<0.001**
	Temperature/ humidity treatment (present)	0.51	0.27	1.90	**0.067**
	*D*. *tasmanica* (present)	0.94	0.29	3.23	**0.003**

In the models exploring the impact of treatments on plant health and soil moisture, the climate treatment had a significant impact on all three, with cages with elevated temperature and reduced humidity having significantly lower plant health and soil moisture (Table 5). Finally, models featuring pests or pathogens as response variables, showed that there was significantly less cover of *Botrytis*, significantly more cover of powdery mildew and significantly fewer LBAM larvae in the simulated climate cages (Table 5). The *D*. *tasmanica* treatment also had a significant positive effect on the cover of powdery mildew.

**Table 5:** Results from mixed effects models. These are testing the effects of the experimental treatments on the pathogens, LBAM, plant health, stress and moisture variables as response for the two years. The treatment variables here are presence/absence factors, and the estimated effect indicates the increase in the response variable in the presence of each treatment.

	Year 1					Year 2				
Response	Explanatory	Est.	s.e.	t	p	Explanatory	Est.	s.e.	t (z)	p
SPAD	Intercept	23.5	0.63	37.40	<0.001	Intercept	19.79	0.43	46.41	<0.001
	Temperature/ humidity	-2.03	0.74	-2.77	**0.009**	Temperature/ humidity	3.67	0.60	6.11	**<0.001**
						Ants	1.37	0.63	2.19	**0.038**
Moisture	Intercept	12.72	0.48	26.59	<0.001					
	Temperature/ humidity	-2.27	0.68	-3.37	**0.002**					
Powdery mildew cover (Year 1: log, Year 2: logit)	Intercept	0.19	0.11	1.59	0.12	Intercept	1.39	0.34	4.05	<0.001
	Temperature/ humidity *D*. *tasmanica*	0.76 0.54	0.15 0.16	5.02 3.42	**<0.001** **0.002**	Temperature/ humidity	1.73	0.49	3.54	**0.002**
*Botrytis* cover (logit Year 2 only)	Intercept	4.27	0.37	11.60	<0.001	Intercept	-1.54	0.23	-6.70	<0.001
	Temperature/ humidity	-1.20	0.48	-2.49	**0.018**	Temperature/ humidity	-2.82	0.24	-11.74	**<0.001**
LBAM	Intercept	1.28	0.16	8.11	<0.001					
	Temperature/ humidity	-0.55	0.22	-2.45	**0.02**					
Sooty Mold *						Intercept	-5.50	2.05	-2.68	0.007
						Mealybug numbers (log)	1.58	0.60	2.64	**0.008**
No of Mealybugs (log)						Intercept Temperature/ humidity Ants	0.79 0.60 1.75	0.23 0.71 0.59	3.37 0.85 2.97	**0.002** 0.405 0.006

*The exception to this is the sooty mold model: the response here is presence/absence, and the test statistic is a z value.

**Fig 2:**
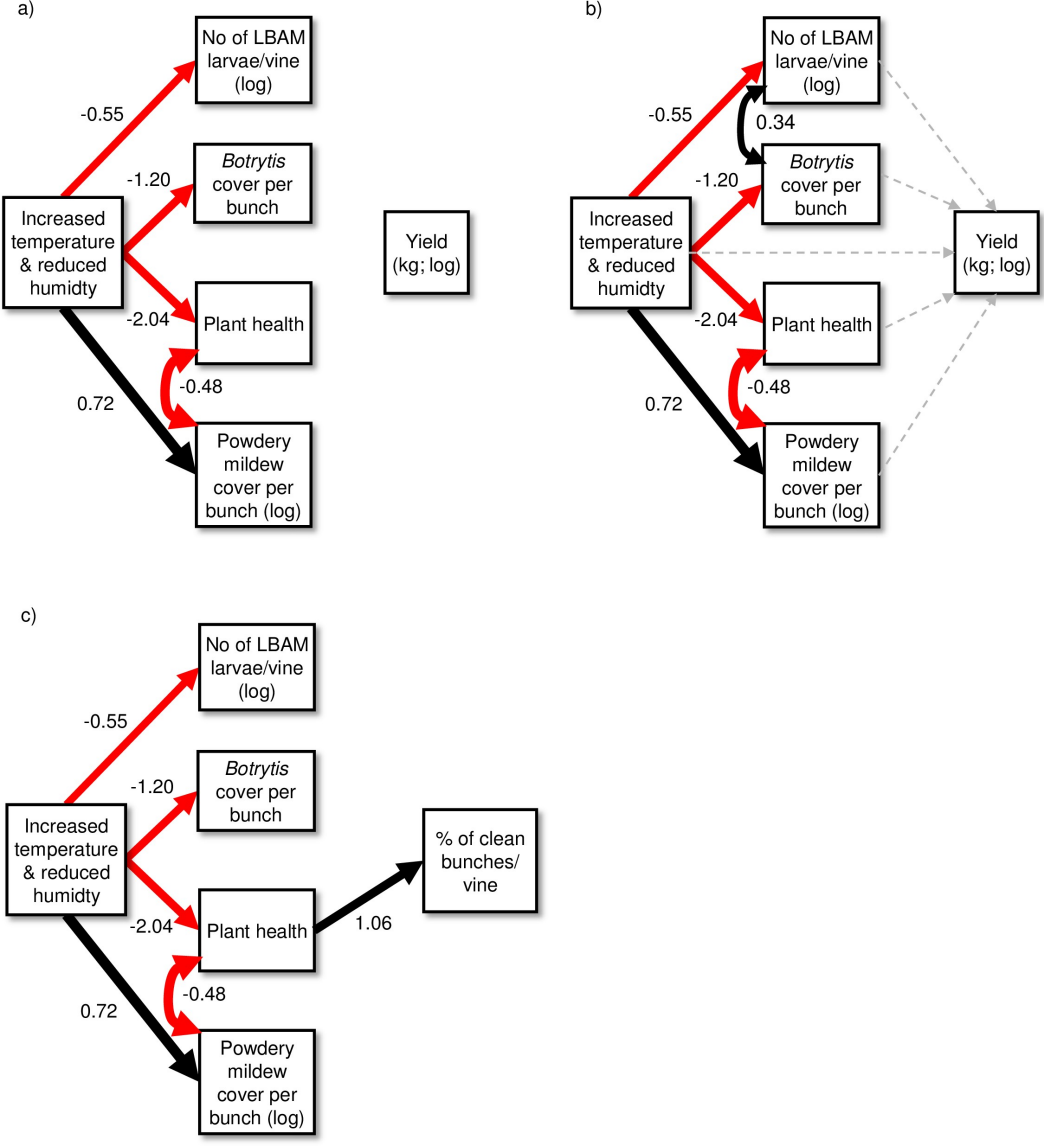
**Path models for the 2009/2010 field season**: a) Second model including only those effects from initial mixed modelling, and the “missing” path between powdery mildew and plant health detected by the tests of directed separation; b) Third model including additional hypothesised paths; c) best fitting model using percentage of clean bunches as focal response variable. Red arrows = negative effects; black arrows = positive effects; and grey, dashed arrows = non-significant relationships (*P*>0.05). Double-headed arrows = correlated errors. Weights of arrows are proportionate to standardized coefficients; numbers beside arrows are unstandardized coefficients. LBAM = light brown apple moth.

The path analysis results are depicted in Fig 2. The first model with Yield as the primary focus and using only those paths suggested by the previous analysis (excluding the *D*. *tasmanica* treatment), was a reasonable fit to the data (Fisher’s C = 26.82, d.f. = 20, p = 0.14; model diagram not shown), and suggested one missing path between powdery mildew and plant health. We added this path as a double headed arrow (i.e. modelled as correlated errors, rather than a causal link), as a causal direction in either direction could be argued and there was a lack of empirical evidence to help with this decision (Fig 2A). The addition of this path improved the model fit (C = 18.02, df = 18, p = 0.455, ΔAICc = -22.64). The third model, including additional hypothesised pathways (Fig 2B), was not an improvement on the second model (C = 5.02, df = 6, p = 0.541, ΔAICc = 43.75). In this analysis, there was no direct impact of the warming treatment on yield, and the total effect of the climate treatment on total yield (direct + indirect effects) from the third model was negligible (standardised coefficient effect: -0.07, unstandardized coefficient effect: -0.05). The same models were applied to the percentage of clean, harvestable fruit, and again the second model was the best fitting (Fig 2C; C = 18.02, df = 18, p = 0.455). However, while there was no direct effect of the climate treatment on percentage of clean bunches, there was a small negative total effect of the treatment on clean yield (standardised coefficients: -0.30, unstandardized coefficients: -2.53). This net effect on clean yield was particularly due to the negative indirect effects of the temperature treatment on plant health, and the increased powdery mildew infection. In general, grape bunches in warmed field cages had increased powdery mildew infection, both due to a direct impact of temperature and an indirect path via declining plant health (measured as leaf chlorophyll). The warming treatment also reduced LBAM abundance and *Botrytis* bunch rot as per the mixed model analysis.

### Year 2

In the second field experiment, there was a treatment effect on total yield (Table 3), with the plastic cover treatment (increased temperature and reduced humidty) having a highly significant positive effect on yield (Table 6). For the models testing the impact of other pressures on yield, there was no effect of plant health, but there was a highly significant negative effect of *Botrytis* bunch rot cover on total yield, and a weaker negative effect of powdery mildew cover on both total yield and the presence of clean bunches (Table 7). The third series of models testing the impacts of treatments on plant health and pests and pathogens showed some contrasting results to Year 1 (Table 5). While there was again a highly significant positive effect of the plastic covers on powdery mildew cover and negative effect on *Botrytis* bunch rot, the climate simulation also positively affected plant health. Mealybugs also contributed to the presence of sooty mold with a significant impact of mealybugs on the occurrence of the pathogen.

**Table 6:** Results from Year 2. Results from a linear mixed effect model of Yield (log transformed) from Year 2 as response and the simulated climate treatment as explanatory variable.

	Estimate	Standard error	t	p
Intercept	2.21	0.06	36.12	<0.001
Increased temperature & reduced humidity	0.31	0.07	4.13	**<0.001**

**Table 7:** Results from mixed effects models, year 2. Result for models of two of the yield variables in Year 2 against pathogen variables. These are the minimum adequate models following backward stepwise selection of full models including all pathogen, pest and plant health variables as explanatory variables. The remaining main effects are those that had a significant effect on the response.

Response	Explanatory	Estimate	s.e.	t (z*)	p
Yield (log)	Intercept	2.06	0.10	20.83	<0.001
	*Botrytis* cover (logit)	-0.16	0.03	-5.14	**<0.001**
	Powdery mildew cover (logit)	-0.07	0.03	-2.28	**0.032**
Presence Clean Bunches*	Intercept	1.04	0.82	1.26	0.209
	Powdery mildew cover (logit)	-0.84	0.37	-2.28	**0.023**

*Percentage clean bunches was converted to presence/absence due to the high number of 0 values, and these results are from a binomial generalised mixed model, where the test statistic is a z value rather than a t-value.

**Fig 3:**
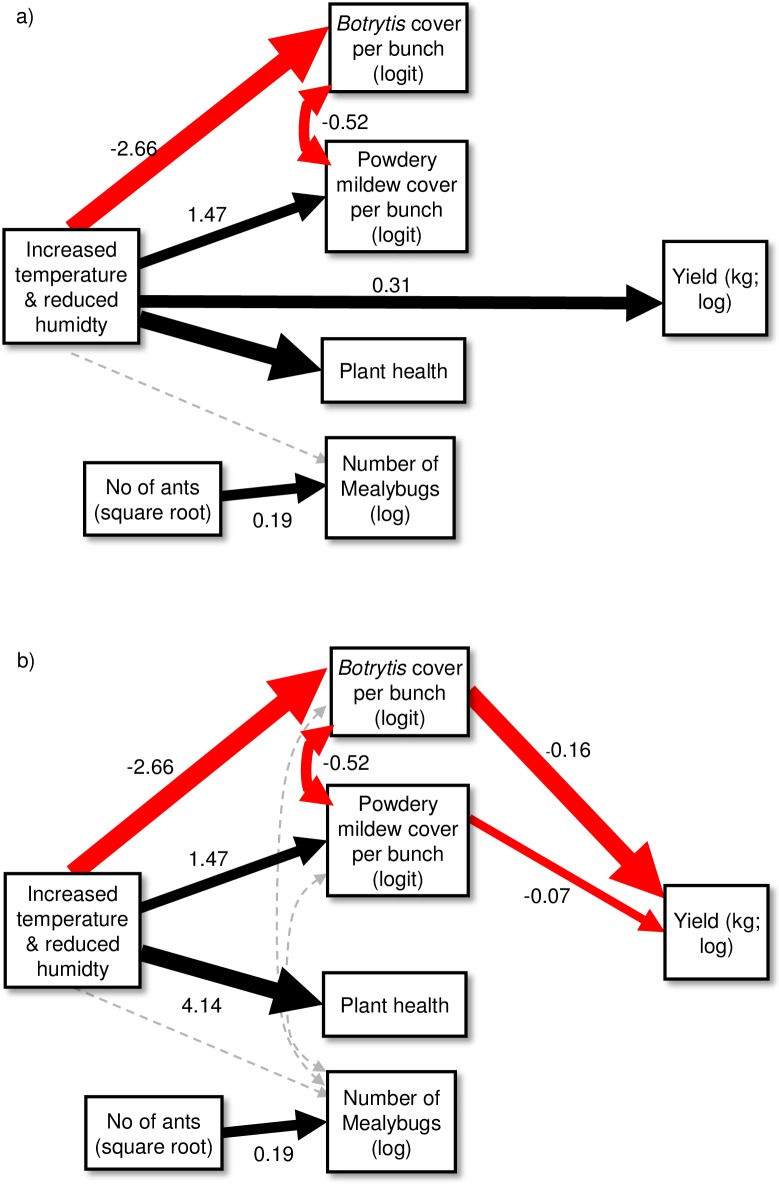
**Path models for the 2010/2011 field season**: a) best fitting model with a direct link between the warming treatment and total yield, b) best fitting model with indirect links between the warming treatment and total yield through the two main pathogens. Red arrows = negative effects; black arrows = positive effects and grey, dashed arrows = non-significant relationships (*P*>0.05). Double-headed arrows represent correlated errors. Weights of arrows are proportionate to standardized coefficients; numbers beside arrows are unstandardized coefficients.

The path analysis also contrasted with the first year (Fig 3), and we found two approaches to structuring the model. Firstly, using the results from the mixed effects models, the warming treatment had a direct positive impact on total yield (standardised coefficient effect: 0.57, unstandardized coefficient effect: 0.31; Fig 3A), as well as direct effects on *Botrytis* cover, powdery mildew cover and plant health. As with the first year, the model with the added “missing” link between *Botrytis* and powdery mildew was the best fitting (C = 29.90, df = 26, p = 0.272, AICc = 564.8). However, we were unable to add the links between *Botrytis* and powdery mildew and Yield due to collinearity issues (Variance Inflation Factors above 3). We therefore explored a second approach, which replaced the direct link between the warming treatment and total yield with indirect links through the two main pathogens (Fig 3B). The best model of this approach included the double-headed arrow and other hypothesised links (C = 23.29, df = 20, p = 0.275, AICc = 708.48). In this model, the net impact of the warming treatment was also positive on total yield and slightly greater than the first approach (standardised coefficient effect: 0.64, unstandardized coefficient effect: 0.35), although the AICc value was higher, indicating a poorer fitting model. Nevertheless, the R^2^ values for the total yield response variable were better for the second approach (Fig 3A: marginal R^2^: 0.32, conditional R^2^: 0.40; Fig 3B: marginal R^2^: 0.43, conditional R^2^: 0.58). We excluded sooty mold cover from the path analysis because the high number of zeros led to convergence issues.

## Discussion

With this study we sought to demonstrate the relative importance of both direct and indirect effects of simulated warming on wine-grape yield. We found contrasting results in two experiments, with simulated warming having a negligible impact on crop yield in the first year, as well as a negative effect on the percentage of non-fungus-infected fruit, and a net positive impact in the second year. These results are partly due to the slightly different food webs used in the experiments, but we also suggest here that the weather conditions in each growing season played an important role.

### Year 1

In the first experiment, there were no strong direct effects of the four treatments on any of the yield measurements, but the climate treatment had an influence on the other organisms in the community, increasing powdery mildew cover and reducing both *Botrytis* bunch rot cover and the abundance of LBAM larvae. Soil moisture and plant health also decreased in the climate treatment. While the exploratory path analysis revealed only negligible net effects of the climate treatment on overall grape yield, there was a net negative effect on percentage of clean berries largely due to the reduced plant health and the increased cover of powdery mildew in this community. These findings can be explained in part in the context of the prevailing weather conditions. The first growing season was characterized by relatively dry weather, particularly in the months of the experiment (February to April, Fig A in S1 Appendix), such that the warmer and even drier conditions in the ‘treatment’ cages were sufficient to decrease plant health, probably due to related effects on soil moisture. This is likely to have left the grapevines more prone to powdery mildew, which would explain the negative effect of plant health on powdery mildew cover, and this may have been confounded by a negative effect of temperature on plant resistance [15]. However, the negative relationship between these two variables was considered “two-way” in the path model (circular paths are not permitted in piecewise SEM) because powdery mildew also infects green tissues of the grapevine plant [44], which would negatively impact plant health as measured by leaf chlorophyll. The positive response of powdery mildew to the temperature treatment probably occurred because this species tends to develop earlier than *Botrytis*, for example, under suitable conditions [45] and can increase development rates at higher temperatures [26]; however, the dry conditions would not have benefitted this species in particular [27]. Conversely, the spread of *Botrytis* was limited by the temperature treatment conditions but also by reductions in LBAM populations, the larvae of which would otherwise have served as a vector for this pathogen. This is supported by the positive correlation between these organisms in the model in Fig 2B. In warm and dry conditions, *Botrytis* spreads less effectively, often via secondary infections in grape berry wounds made by insects [28], so fewer LBAM larvae would have negatively impacted the pathogen. The temperatures in the climate treatment cages may also have risen above optimal thresholds for the LBAM, particularly at the vulnerable egg and first instar stages [25], reducing populations.

### Year 2

In the second experiment, the greater total yield in cages with plastic covers was the only direct treatment effect, potentially due to poor responses of introduced insects over the experimental period. The climate treatment also had a positive impact on plant health, in contrast to the first experiment. However, this did not apparently improve resistance to pathogens, as there were high infection rates of both powdery mildew and *Botrytis* infection in this season, and the link between plant health and clean bunches was not found. Nevertheless, the impact of the climate treatment on these pathogens was in agreement with the first year: powdery mildew increased and *Botrytis* decreased in response to higher temperatures and reduced humidity. This supports the previous year’s results, suggesting an early advantage of powdery mildew and the sensitivity of *Botrytis* to temperature. Despite the high rates of infection in this experiment, the second approach to the path analysis (Fig 3B) suggests that the main contributor to the net increased yield under warmer and drier conditions is the reduction in *Botrytis* infection in cages with plastic covers, both directly as a result of the treatment, but also via a possible antagonistic interaction with powdery mildew. This negative interaction is not supported by other empirical evidence, however, as powdery mildew infection often leads to bunch rotting fungi such as *Botrytis* [44, 46]. The negative link between the two pathogens may instead indicate that powdery mildew infection increases when *Botrytis* is unable to spread sufficiently broadly (i.e., low *Botrytis* cover creates more space for powdery mildew infection). The increase in powdery mildew in treatment cages has a contrasting but smaller net effect on yield, and this supports previous findings [47, 48]. Unfortunately, we could not disentangle the direct and indirect effects of the climate treatment on total yield, but these interactions warrant further investigation, particularly as the two path analysis approaches suggest complex pathways involved in the response of this system to environmental changes.

Our data did not suggest an increase in mealybug numbers under warmer conditions, but the positive effect of the presence of ants and the mealybug impact on sooty mold presence occurred as expected. Furthermore, the path model analysis suggests that these effects occurred with limited impact on the other organisms. We did not find support for previous evidence that ants or mealybugs facilitate *Botrytis* infection [28], for example. This suggests that both mealybugs and sooty mold may benefit from future climate changes, but not to the extent that they impact yield.

Taken together, the second year’s results can also be explained in part by the prevailing weather conditions. Growing conditions were damper with slightly lower than normal temperatures towards the end of the season (Fig A in S1 Appendix), probably elevating soil moisture in all plots (although this was not measured). Vines would therefore have benefitted more from the induced warmer growing conditions, promoting more flowering and higher growth rate without suffering from the water stress of the first season [12]. However, while yield and plant health benefited from the conditions and temperature treatment, there were very few clean bunches overall, with high rates of both powdery mildew and *Botrytis* infection. These two pathogens are also likely to have benefited from the moister conditions. *Botrytis* in particular spreads well in those conditions [28, 29], and powdery mildew infection is generally more severe during cooler and more humid years [44, 49].

### Implications

The results from the two seasons indicate that the effect of future increases in temperature on yield and, as far as we have been able to test, the percentage of clean grapes, will depend in part on precipitation levels during the growing season. For this region of New Zealand, most climate models predict a decrease in summer precipitation [31], suggesting that the impacts demonstrated in the first field season are more likely to prevail in the future. If this is the case, without additional management, these results suggest that yield will be unaffected, *Botrytis* and LBAM will reduce, but plant health will also suffer, potentially reducing the clean bunch harvest through increased powdery mildew infection, although the incidence of this fungus is considered likely to decline with climate change [49]. Increases in the frequency and magnitude of both extreme precipitation events and dry spells are also expected [31], however, making future predictions extremely difficult. In addition, the scenario above is obviously a simplification of reality, as many other organisms may impact grape yield simultaneously, and we have not tested the combined impact of mealybugs and LBAM for example. Nevertheless, this research has shown the range of direct and indirect pathways through which a) weather conditions, b) organism interactions and c) plant health combine to affect the yield of marketable grapes under simulated climate change treatments. Our results demonstrate that predictions concerning the impacts of climate change on crops need to account for the complexity of agro-ecological communities and multi-species antagonistic and facilitative interactions. Models constructed with individual or pairwise species responses are unlikely to yield realistic results, as these may be modified by indirect effects in multispecies interaction networks [50]. These effects can include trait-mediated, non-consumptive effects [51], although these do not appear to be operating in this work.

Our findings also demonstrate that more research is needed to improve future crop management recommendations. While farmers can have little influence over climate change or weather conditions individually, they can partially mitigate abiotic impacts through better management of plant health and understanding the dynamics of agro-ecological communities. Therefore, future research should be directed at identifying the most important local pests and diseases and the results of their interactions under varying simulated weather conditions. Recommendations can then aim to interpret the impact of probable future weather conditions and identify situations when plant health requires more intense management, for example. Similarly, while grapevines are commonly put under mild water stress to improve grape yields and wine quality [12], this research demonstrates that over-stressed vines can be more susceptible to certain pest and disease problems.

We have not considered the impact of other climate drivers such as elevated CO_2_. This can affect the plant directly and alter plant C:N ratios, reducing the nutritional quality of the plant for herbivores and requiring them to consume more leaf material to meet energy requirements [5]. Adding more natural enemies to our system would also incorporate additional indirect pathways and would make our findings more realistic in relation to the open field. Unfortunately, our attempts to include these were not successful, as survival rates of the enemies were low across all treatments. However, predators and parasitoids may perform better or worse under climate change, with subsequent influences on pest populations [4, 25]. Nevertheless, as we better understand the change in dynamics between species in food webs under changing climate, we draw closer to better anticipating the effects of these changes on food supply and its quantity in the future.

## Supporting information

S1 Appendix Methods details. Filename “S1 Appendix.docx”: Full details of methods, Tables of path analysis results and Fig A: Mean monthly temperatures and precipitation for the study area.
(DOCX)

S1 File Data from the experiments used in the analyses. (XLSX)
