# Supplementary material for: Community dynamics can modify the direction of simulated warming effects on crop yield
Source: PLoS One. 2018 Nov 19;13(11):e0207796. doi: 10.1371/journal.pone.0207796 (PMC6242358; doi:10.1371/journal.pone.0207796)
Supplement: S1 Appendix — Filename “S1 Appendix.docx”: Full details of methods, Tables of path analysis results and Fig A: Mean monthly temperatures and precipitation for the study area. (DOCX) [file pone.0207796.s001.docx]

**Supporting information: S1 Appendix**

**Title**: Community dynamics can reverse the direction of simulated warming effects on crop yield

**Authors**: Mark A. K. Gillespie, Marco Jacometti, Jason M. Tylianakis, Steve D. Wratten

**Full details of Methods**

***Site description (both years)***

The experiment was conducted in a research vineyard at Lincoln University, Canterbury, New Zealand (43°38 S, 172°27 E, 9 m ASL). The grapes were unirrigated, 16 year old Chardonnay Mendoza, on their own rootstocks. They were spur pruned and erected on a vertical shoot positioned (VSP) trellis system. Prior to the experiments, the vines had been managed to commercial standards, receiving two botryticide sprays per season and herbicide and sulfur applications as required. The vines were spur-pruned on July 2 2009 and July 7 2010 and head trained on September 10 2009 and September 7 2010 to achieve a “goblet” vine training system independent of trellis ^1^. This system was adopted so that the trellis could be removed to allow the field cages (see below) to be placed over the vines without impediments. The collection and culturing of insects from both years is described below. Botrytis and sooty moulds occurred naturally, depending on the year of study.

## Sources, collection and rearing of invertebrates

*Year 1*

In year 1, the field experiment used light brown apple moth (LBAM) larvae and adults of the parasitoid wasp *Dolichogenidea tasmanica* (Cameron) (Hymenoptera: Braconidae) and the European earwig (*Forficula auricularia* L. Dermaptera: Forficulidae) a general predator. However, the earwigs and parasitoids did not perform well in the field cages, had no effect on any other variables and were disregarded in final data analyses. Nevertheless, the source and culture of all three are described here.

Batches of light brown apple moth eggs of even age were purchased from Plant and Food Research Limited, Mt Albert, New Zealand. They were received on sheets of A4 paper, which were cut into 2 × 2 cm squares, each of which contained between 55 and 65 eggs. These eggs were used directly in the field cage experiments

To collect the parasitoid wasp *D. tasmanica,* further batches of LBAM were purchased from the same supplier. The eggs were again received on sheets of paper which were cut into 2 x 2cm squares so that each square held at least one egg batch of more than 50 eggs. When these were within 2-4 days of hatching, as indicated by a darkening in color, they were transported to various vineyard blocks. These were Milton Vineyard and Winery, Gisborne, New Zealand on October 16 2009 and November 9 2009 (Hill Block, 38°41 S, 177°51 E, 42 m above sea level (ASL); and Vineyard Block, 38°40 S, 177°53 E, 0 m ASL), and to both Wither Hills Vineyard, Marlborough, New Zealand (Rarangi Block, 41°24 S, 174°01 E, 0 m ASL; and Old Road Block, 41°30 S, 173°54 E, 12 m ASL) and Mud House Winery and Café, Waipara, Canterbury, New Zealand (Block K, 43°04 S, 172°45 E, 60 m ASL) on October 19 and November 5 2009. At these locations, the egg batches were each stapled to the abaxial surface of a single leaf at the end of a shoot from grape vines of various varieties and ages. This shoot was then covered for one week with 15 × 30 cm mesh bags, with a mesh size of 0.3 × 0.3 mm, open at one end and tied with cable ties, then uncovered to allow recently-hatched larvae to be parasitized by *Dolichogenidea tasmanica* (Cameron) (Hymenoptera: Braconidae) for a period of ten days, following the protocol of Berndt ^2^. The vine shoot was then removed and the LBAM larvae were individually placed in capped 15 mL plastic tubes and fed on an artificial diet modified from Singh ^3^. They were then left to develop under laboratory conditions. As *D. tasmanica* individuals emerged, they were transferred every three days into capped 25 × 15 × 15 cm plastic containers, so the age of individuals would be known to an accuracy of three days. They were then fed on a 50% honey solution until they were required for the field experiment.

Adults of European earwig (*Forficula auricularia*) (Linn.) (Dermaptera: Forficulidae) were collected from November 2 to December 7 2009 using corrugated cardboard rolls wound around the trunks of nectarine trees (*Prunus persica* (Batsch)) as described by Burnip et al., ^4^ in an abandoned orchard in the Biological Husbandry Unit, at Lincoln University (43°39 S, 172°27 E, 8 m ASL). This orchard was overgrown with vegetation and had not received any agrichemical sprays or treatment for 18 years. Once collected, the earwigs were placed in rectangular plastic containers (30×20×15 cm), half filled with bark chip mulch, and sealed with plastic lids with a mesh centre. The earwigs were fed a diet of vegetables and cat food, the remnants of which were removed before fungi could develop. Earwigs were repeatedly collected and added to this container until the population was sufficient for subsequent release into field cages.

*Year 2*

Cultures of the citrophilus mealybug, (*Pseudococcus calceolariae*) (Maskell) (Hemiptera*:* Pseudococcidae) were purchased from Zonda Resources Limited, Pukekohe New Zealand and maintained on sprouting potatoes (*Solanum tuberosum,* various varieties) in 30 × 20 × 10 cm plastic containers. Each container had six 3cm holes drilled in the lid, which was covered with 0.3 × 0.3 mm mesh, and was kept in a climatically controlled room (18°C +/- 2°C; 16:8 h (L:D) photoperiod). This temperature was selected to promote rapid reproduction ^5^. Every two weeks, the mealybugs from each box were evenly distributed into two other similar containers and new sprouting potatoes were added. This was done to encourage rapid population growth, and populations were regularly assessed until they were sufficient for subsequent release into field cages.

Southern ants (*Monomorium antarcticum*) (Smith) (Hymenoptera: Formicidae) were collected by quickly excavating wild colonies, ensuring that at least 50% of the brood and at least one queen were obtained. They were collected from two locations in Lyttelton, Canterbury, New Zealand (43°36'10.06"S, 172°43'25.91"E, 44 m ASL) and 43°36'4.88"S, 172°43'22.36"E, 62m ASL), and one in Charteris Bay, Canterbury, New Zealand (43°38'39.29"S, 172°42'42.16"E, 10 m ASL). Each colony was collected with the surrounding soil in a 40 × 40 × 15 cm plastic container. Each container was completely filled. They were then immediately transported to the field site where they were introduced into the appropriate field cages.

## Experimental design

## Field season 1 (July 2009- April 2010)

In September 2009, 36 field cages were erected over individual vines. Each cage had a basal area of 1.8 x 1.8 m and was 2.0 m high. Cages were approximately 2 m apart and constructed of a metal frame covered with a net (mesh size: 0.28 x 0.78 mm). Half of the cages had a transparent polyethylene cover placed over the top, also covering the upper 1 m of the cage sides. These covers reduced heat convection and prevented direct rainfall and therefore reduced cage relative humidity (see Table S1).

Table A. Mean and standard error of the temperature and humidity in cages with and without plastic covers in each year, with confidence intervals (CI) and results of a t-test for the differences.

|  |  |  | **Mean** | **Standard Error** | **Difference** | **Confidence Intervals** | **t** | **p** |
| --- | --- | --- | --- | --- | --- | --- | --- | --- |
| **Year 1** | **Temperature (°C)** | Plastic | 17.3 | 0.08 | 0.8 | 0.65 ; 1.10 | 8.01 | <0.001 |
|  |  | No plastic | 16.5 | 0.08 |  |  |  |  |
|  | **Humidity (%)** | Plastic | 73.5 | 0.26 | -1.7 | -2.34 ; -1.16 | -6.16 | <0.001 |
|  |  | No plastic | 75.2 | 0.12 |  |  |  |  |
| **Year 2** | **Temperature (°C)** | Plastic | 13.8 | 0.16 | 1.1 | 0.76 ; 1.48 | 6.53 | <0.001 |
|  |  | No plastic | 12.7 | 0.06 |  |  |  |  |
|  | **Humidity (%)** | Plastic | 79.9 | 0.59 | -3.5 | -4.78 ; -2.20 | -5.73 | <0.001 |
|  |  | No plastic | 83.4 | 0.16 |  |  |  |  |

Humidity and temperature data were measured in each cage every 15 minutes with Hygrochron™ iButton DS1923 dataloggers (maxim integrated). These covered cages produced conditions predicted by climate change models for the study region ^6^, and are hereafter called the “warming treatment”. The vines within the cages were then treated with the insecticide dichlorvos (Nuvos^TM^, 0.175% 2,2‑dichlorovinyl dimethyl phosphate, 1-1.5L per cage) to kill all resident arthropods, then left for six days. Dichlorvos was chosen for this purpose because of its broad-spectrum activity ^7^ and short persistence in the environment (95% reduction in concentration in the first 20 minutes ^8^). All cages were arranged in a 3-block/replicate, 2 × 2 x 2 x 2 factorial, randomized block design, with twelve treatment combinations comprising the presence or absence of plastic covers, LBAM, *D. tasmanica* and earwigs with the exception that *D. tasmanica* was never added when its host LBAM was absent*.* On December 21 2009, four batches each of between 55 and 65 LBAM eggs laid on paper were stapled to the underside of four vine leaves, approximately one meter from the ground and equally spaced around the perimeter of each of the vines in the LBAM cages. On January 1 2010, four pairs (male and female) of adult *D. tasmanica* aged between 4 and 12 days, and four pairs of earwigs of equal weight, were added to the appropriate cages. Any arthropods, other than those species introduced to the cages, that had entered the cages since the dichlorvos spraying were recorded and then killed by hand on a weekly basis. On February 8 2010, the vines were sprayed with Systhane^TM^ 400 WP (0.4% myclobutanil, 1-1.5L per cage) with a knapsack sprayer to control powdery mildew. This was done so that incipient mildew infections were reduced uniformly to very low levels at the start of the experiment. This fungicide was chosen as it has no effect on *B. cinerea* or the insect species in this experiment.

## Sampling

On April 9 2010, a sample of ten grape bunches was removed from adjacent vines, of the same variety and history as the vines used in this experiment, and assessed for sugar concentrations with an optical refractometer to assess if the grapes were ready to be harvested. At this time, the mean sugar concentration was 20.7 °Brix and considered ready for harvest. Over the following week, soil moisture, vine stress, chlorophyll content, yield, berry set, botrytis bunch rot and powdery mildew infection, LBAM and *D. tasmanica* abundance and damage and earwig populations were measured in the cages as follows. On 12 April, soil moisture was measured with an electronic soil moisture meter (Campbell Scientific “Hydrosense” hand held display CD620 and CS620 12 cm probe) in four locations per cage, each approximately 30 cm from the vine trunk and equally spaced around the vine. The measurements were made between 10.00-11.30 h to allow dew to have evaporated. On March 8, pre-dawn water pressure was measured between 03.00-04.30 h following the protocol described in Hofmann *et al.* ^9^. For this, four young leaves were selected per vine from its perimeter, at a height of approximately 1 m. These measurements were made *in situ* and within 1 minute of the leaf’s being sampled. On March 8, leaf chlorophyll content was measured with a SPAD (Soil Plant Analysis Development) meter (Konica Minolta SPAD-502 (Osaka, Japan)) on twenty young leaves per vine from the top and sides of the vines’ outer foliage. SPAD was used as it is a good indicator of water stress ^1,10^ and temperature stress ^11,12^. On March 9, the grape bunches were removed from the vines, then weighed and searched for LBAM larvae, LBAM damage, berry set and botrytis bunch rot and/or powdery mildew infection. Three days later, all the leaves were removed from the vines and each leaf was assessed for the presence of LBAM and *D. tasmanica* and damage caused by LBAM. Earwigs were counted and recorded in each cage using corrugated cardboard rolls that had been placed around the trunks of the vines following the methodology used in ^4^.

## Field season 2 (July 2010- April 2011)

## Experimental design

On September 2010, 32 of the same field cages used in the previous year were erected over different individual grape vines from the ones used in the previous year, but of the same variety and from the same vineyard block. All cages were arranged in a 4‑block/replicate, factorial, randomized block design, with eight treatments comprising the presence or absence of plastic covers, ants and mealybugs. On January 19 2010, a potato (cv. Désirée) slice with approximately 60 mealybugs, of mixed age and sex from a laboratory culture, was attached with cable tie to a single grape bunch in the middle of the canopy on the northern (warmer) side of each vine. This bunch was then covered for one week with 15 × 30 cm mesh bags, with a mesh size of 0.3 × 0.3 mm, open at one end and tied with cable ties. This allowed the mealybugs to move from the dehydrating potato slice into the bunches and establish without the threat of predation or their moving to other parts of the plant or falling to the ground before selecting a vine feeding site.

From January 26-28 2010, ant colonies were introduced into the appropriate cages, one block per day over the three successive days. Colonies were introduced to the cages on the same day that they were collected to minimize stress on the insects. The plastic boxes in which the colony had been collected then had four-15 mm holes drilled into the bottom corners of each to facilitate drainage. A further three similar holes were drilled in the top section of each of the outer walls to allow ants access to the cage. The soil level in the container was then checked to be in contact with the lid and then a single 25 × 25 × 7.5 cm black concrete paving stone was placed on top of the lid. This was used to regulate the temperature of the colony by absorbing heat over the day and conducting it at night. Two cylindrical 60 ml plastic containers with screw-top lids with 50 mm long × 8 mm diameter cylindrical wicks through their centers were placed on top of the paving stones, one containing a 50% honey solution, the other containing water. Approximately 20g of moist dog food, as a protein source, was also placed on the stone. For the first three weeks the food, honey solution and water containers were replaced weekly. For the following six weeks they were still replaced but reduced in amount weekly. For this time, the honey was placed in an undiluted form directly on the paving stone. It was applied in this way as it was usually completely consumed early in the week and the ants then foraged independently for the remainder of the period. This allowed for a more effective transition to being completely independent for the later parts of the experiment. By April 1 2011, no supplemental food was added to the cages.

As in the previous year, any arthropods, other than those species introduced to the cages, that had entered the cages since the dichlorvos application, were recorded and then killed by hand on a weekly basis. On February 29 2011, the vines were sprayed with Systhane^TM^ 400 WP (0.4% myclobutanil, 1-1.5L per cage) with a knapsack sprayer to manage powdery mildew. As in the previous season, this fungicide was chosen as it has no effect on *B. cinerea*, sooty mould or the other insect species in this experiment.

## Sampling

On April 22 2011, following the protocol used in the previous year, bunches from grape vines adjacent to the field cages were tested for sugar concentration and considered ready for harvest when a mean of 22.4 °Brix was reached. Over the following week, leaf chlorophyll content, vine yield, berry set, botrytis bunch rot and powdery mildew infection were measured according to the protocol followed in the previous year. Sooty mould infection on bunches was assessed using the same protocol as for the other plant pathogens. Mealybug populations were assessed by counting individuals in bunches. Ant colonies were excavated then separated into colonized and non-colonized soil. The volume of the colonized soil was then measured in 300 mL beakers. This was done on a cool morning, as at this time the colony was less active and most of the workers would still have been within the colony.

**References**

1 Jackson, R. S. *Wine Science: Principles and Applications*. (Academic Press, 1994).

2 Lowrance, R., Dabney, S. & Schultz, R. Improving water and soil quality with conservation buffers. *Journal of Soil and Water Conservation* **57**, 36A-43A (2002).

3 Singh, P. A general purpose laboratory diet mixture for rearing insects. *Insect science and application* **4**, 357-362 (1983).

4 Burnip, G. M., Daly, J. M., Hackett, J. K. & Suckling, D. M. European earwig phenology and effect of understorey management on population estimation. *New Zealand Plant Protection* **55**, 390-395 (2002).

5 Charles, J. G., Froud, K. J. & Henderson, R. C. Morphological variation and mating compatibility within the mealybugs *Pseudococcus calceolariae* and *P. similans* (Hemiptera: Pseudococcidae), and a new synonymy. *Systematic Entomology* **25**, 285-294 (2000).

6 Reisinger, A., Mullan, B., Manning, M., Wratt, D. & Nottage, R. in *Climate Change Adaptation in New Zealand* (eds R. Nottage, D. Wratt, J. Bornman, & K. Jones) 26-43 (New Zealand Climate Change Centre, 2010).

7 Young, S. *New Zealand Agrichemical manual*. 779 (Agri Media Ltd, 2009).

8 Casida, J. E., McBride, L. & Niedermeier, R. P. Metabolism of 2,2-dichlorovinyl dimethyl phosphate in relation to residues in milk and mammalian tissues. *Journal of Agricultural and Food Chemistry* **10**, 370-376 (1962).

9 Hofmann, R. W. *et al.* Responses to UV-B radiation in Trifolium repens L. - physiological links to plant productivity and water availability. *Plant Cell and Environment* **26**, 603-612 (2003).

10 Jangpromma, N., Songsri, P., Thammasirirak, S. & Jaisil, P. Rapid assessment of chlorophyll content in sugarcane using a SPAD chlorophyll meter across different water stress conditions. *Asian Journal of Plant Sciences* **9**, 368-374 (2010).

11 Taub, D. R., Seemann, J. R. & Coleman, J. S. Growth in elevated CO2 protects photosynthesis against high-temperature damage. *Plant Cell and Environment* **23**, 649-656 (2000).

12 Baninasab, B. & Ghobadi, C. Influence of Paclobutrazol and application methods on high-temperature stress injury in cucumber seedlings. *Journal of Plant Growth Regulation* **30**, 213-219, doi:10.1007/s00344-010-9188-2 (2011).

**Table B:** 2009/10 Field season path analysis output, with percentage of clean berries as the main response variable of interest. Model 1 – including only those paths suggested by intial data analysis with linear mixed modelling. LBAM = light brown apple moth, SPAD = value from the Soil Plant Analysis Development meter. Other terms in parentheses refer to data transformation applied to the variable.

| **Predictor variable** | **Response variable** | **Unstandardised path coefficient** | **Standard error** | **Standardised Path coefficient** | **p-value** |
| --- | --- | --- | --- | --- | --- |
| Plant Health (SPAD) | % clean bunches | 1.06 | 0.37 | 0.44 | 0.007 |
| Increased temperature & reduced humidity | Plant health (SPAD) | -2.04 | 0.74 | -0.42 | 0.009 |
| Increased temperature & reduced humidity | Powdery mildew cover per bunch (log) | 0.72 | 0.18 | 0.53 | <0.001 |
| Increased temperature & reduced humidity | Botrytis cover per bunch | -1.20 | 0.48 | -0.39 | 0.018 |
| Increased temperature & reduced humidity | Number of LBAM larvae | -0.55 | 0.22 | -0.39 | 0.020 |

**Table C:** 2009/10 Field season path analysis output, with percentage of clean berries as the main response variable of interest.. Model 2 – as Model 1, but including the path between Plant Health and Powdery Mildew (in *italics*). LBAM = light brown apple moth, SPAD = value from the Soil Plant Analysis Development meter. Other terms in parentheses refer to data transformation applied to the variable.

| **Predictor variable** | **Response variable** | **Unstandardised path coefficient** | **Standard error** | **Standardised Path coefficient** | **p-value** |
| --- | --- | --- | --- | --- | --- |
| Plant Health (SPAD) | % clean bunches | 1.06 | 0.37 | 0.44 | 0.007 |
| Increased temperature & reduced humidity | Plant health (SPAD) | -2.04 | 0.74 | -0.42 | 0.009 |
| Increased temperature & reduced humidity | Powdery mildew cover per bunch (log) | 0.72 | 0.18 | 0.53 | <0.001 |
| Increased temperature & reduced humidity | Botrytis cover per bunch | -1.20 | 0.48 | -0.39 | 0.018 |
| Increased temperature & reduced humidity | Number of LBAM larvae | -0.55 | 0.22 | -0.39 | 0.020 |
| *~~Plant health (SPAD)* | *~~Powdery mildew cover per bunch (log)* | *-0.49* | *NA* | *-0.49* | *0.001* |

**Table D:** 2009/10 Field season path analysis output, with percentage of clean berries as the main response variable of interest. Model 3 – as Model 2, but including the additional hypothesized paths (in *italics*). LBAM = light brown apple moth, SPAD = value from the Soil Plant Analysis Development meter. ~~ indicates that the pathway represents correlated errors. Other terms in parentheses refer to data transformation applied to the variable.

| **Predictor variable** | **Response variable** | **Unstandardised path coefficient** | **Standard error** | **Standardised Path coefficient** | **p-value** |
| --- | --- | --- | --- | --- | --- |
| *Increased temperature & reduced humidity* | *% clean bunches* | *1.42* | *2.38* | *0.12* | *0.555* |
| *Number of LBAM larvae* | *% clean bunches* | *1.21* | *1.58* | *0.15* | *0.449* |
| *Powdery mildew cover per bunch (log)* | *% clean bunches* | *-1.73* | *2.12* | *-0.20* | *0.422* |
| *Botrytis cover per bunch* | *% clean bunches* | *-0.98* | *0.79* | *-0.26* | *0.225* |
| Plant Health (SPAD) | % clean bunches | 0.82 | 0.54 | 0.34 | 0.144 |
| Increased temperature & reduced humidity | Plant health (SPAD) | -2.04 | 0.74 | -0.42 | 0.009 |
| Increased temperature & reduced humidity | Powdery mildew cover per bunch (log) | 0.72 | 0.18 | 0.53 | <0.001 |
| Increased temperature & reduced humidity | Botrytis cover per bunch | -1.20 | 0.48 | -0.39 | 0.018 |
| Increased temperature & reduced humidity | Number of LBAM larvae | -0.55 | 0.22 | -0.39 | 0.020 |
| *~~Number of LBAM larvae* | *~~Botrytis cover per bunch* | *0.34* | *NA* | *0.34* | *0.024* |
| *~~*Plant health (SPAD) | *~~*Powdery mildew cover per bunch (log) | -0.49 | NA | -0.49 | 0.001 |
| *~~Powdery mildew cover per bunch (log)* | *~~Botrytis cover per bunch* | *-0.14* | *NA* | *-0.14* | *0.219* |

**Table E:** 2009/10 Field season path analysis output, with total yield as the main response variable of interest. Model 1 – including only those paths suggested by intial data analysis with linear mixed modelling. LBAM = light brown apple moth, SPAD = value from the Soil Plant Analysis Development meter. Other terms in parentheses refer to data transformation applied to the variable.

| **Predictor variable** | **Response variable** | **Unstandardised path coefficient** | **Standard error** | **Standardised Path coefficient** | **p-value** |
| --- | --- | --- | --- | --- | --- |
| Plant Health (SPAD) | Yield (log) | 0.04 | 0.03 | 0.27 | 0.116 |
| Increased temperature & reduced humidity | Plant health (SPAD) | -2.04 | 0.74 | -0.42 | 0.009 |
| Increased temperature & reduced humidity | Powdery mildew cover per bunch (log) | 0.72 | 0.18 | 0.53 | <0.001 |
| Increased temperature & reduced humidity | Botrytis cover per bunch | -1.20 | 0.48 | -0.39 | 0.018 |
| Increased temperature & reduced humidity | Number of LBAM larvae | -0.55 | 0.22 | -0.39 | 0.020 |

**Table F:** 2009/10 Field season path analysis output, with total yield as the main response variable of interest. Model 2 – as Model 1, but including the path between Plant Health and Powdery Mildew (in *italics*). LBAM = light brown apple moth, SPAD = value from the Soil Plant Analysis Development meter. Other terms in parentheses refer to data transformation applied to the variable.

| **Predictor variable** | **Response variable** | **Unstandardised path coefficient** | **Standard error** | **Standardised Path coefficient** | **p-value** |
| --- | --- | --- | --- | --- | --- |
| Plant Health (SPAD) | Yield (log) | 0.04 | 0.03 | 0.27 | 0.116 |
| Increased temperature & reduced humidity | Plant health (SPAD) | -2.04 | 0.74 | -0.42 | 0.009 |
| Increased temperature & reduced humidity | Powdery mildew cover per bunch (log) | 0.72 | 0.18 | 0.53 | <0.001 |
| Increased temperature & reduced humidity | Botrytis cover per bunch | -1.20 | 0.48 | -0.39 | 0.018 |
| Increased temperature & reduced humidity | Number of LBAM larvae | -0.55 | 0.22 | -0.39 | 0.020 |
| *~~Plant health (SPAD)* | *~~Powdery mildew cover per bunch (log)* | *-0.48* | *NA* | *-0.48* | *0.002* |

**Table G:** 2009/10 Field season path analysis output, with total yield as the main response variable of interest. Model 3 – as Model 2, but including the additional hypothesized paths (in *italics*). LBAM = light brown apple moth, SPAD = value from the Soil Plant Analysis Development meter. ~~ indicates that the pathway represents correlated errors. Other terms in parentheses refer to data transformation applied to the variable.

| **Predictor variable** | **Response variable** | **Unstandardised path coefficient** | **Standard error** | **Standardised Path coefficient** | **p-value** |
| --- | --- | --- | --- | --- | --- |
| *Increased temperature & reduced humidity* | *Yield (log)* | *0.09* | *0.18* | *0.10* | *0.636* |
| *Number of LBAM larvae* | *Yield (log)* | *0.14* | *0.12* | *0.24* | *0.258* |
| *Powdery mildew cover per bunch (log)* | *Yield (log)* | *0.02* | *0.16* | *0.03* | *0.922* |
| *Botrytis cover per bunch* | *Yield (log)* | *-0.05* | *0.06* | *-0.20* | *0.371* |
| Plant Health (SPAD) | Yield (log) | 0.05 | 0.04 | 0.28 | 0.259 |
| Increased temperature & reduced humidity | Plant health (SPAD) | -2.04 | 0.74 | -0.42 | 0.009 |
| Increased temperature & reduced humidity | Powdery mildew cover per bunch (log) | 0.72 | 0.18 | 0.53 | <0.001 |
| Increased temperature & reduced humidity | Botrytis cover per bunch | -1.20 | 0.48 | -0.39 | 0.018 |
| Increased temperature & reduced humidity | Number of LBAM larvae | -0.55 | 0.22 | -0.39 | 0.020 |
| *~~Number of LBAM larvae* | *~~Botrytis cover per bunch* | *0.34* | *NA* | *0.34* | *0.024* |
| *~~Powdery mildew cover per bunch (log)* | *~~Botrytis cover per bunch* | *-0.14* | *NA* | *-0.14* | *0.219* |
| *~~Plant health (SPAD)* | *~~Powdery mildew cover per bunch (log)* | *-0.48* | *NA* | *-0.48* | *0.002* |

**Table H:** 2010/11 Field season path analysis output, with total yield as the main response variable of interest. Model 1 – including only those paths suggested by intial data analysis with linear mixed modelling. SPAD = value from the Soil Plant Analysis Development meter. Other terms in parentheses refer to data transformation applied to the variable. Model statistics: Fishers C = 39.22, df = 28, p = 0.078, AICc = 632.32

| **Predictor variable** | **Response variable** | **Unstandardised path coefficient** | **Standard error** | **Standardised Path coefficient** | **p-value** |
| --- | --- | --- | --- | --- | --- |
| Increased temperature & reduced humidity | Yield (log) | -0.31 | 0.08 | 0.57 | <0.001 |
| Increased temperature & reduced humidity | Plant health (SPAD) | 4.14 | 0.60 | 0.51 | <0.001 |
| Increased temperature & reduced humidity | Powdery mildew cover per bunch (logit) | 1.47 | 0.44 | 0.51 | 0.003 |
| Increased temperature & reduced humidity | Botrytis cover per bunch (logit) | -2.66 | 0.23 | -0.92 | <0.001 |
| Number of ants (square root) | Number of mealybugs (log) | 0.19 | 0.06 | 0.52 | 0.002 |
| Increased temperature & reduced humidity | Number of mealybugs (log) | 0.49 | 0.73 | 0.12 | 0.512 |

**Table I:** 2010/11 Field season path analysis output, with total yield as the main response variable of interest. Model 2 – as Model 1, but including the covariance path between Botrytis and Powdery Mildew (in *italics*). SPAD = value from the Soil Plant Analysis Development meter. ~~ indicates that the pathway represents correlated errors. Other terms in parentheses refer to data transformation applied to the variable. Model statistics: Fishers C = 29.90, df = 26, p = 0.272, AICc = 564.76

| **Predictor variable** | **Response variable** | **Unstandardised path coefficient** | **Standard error** | **Standardised Path coefficient** | **p-value** |
| --- | --- | --- | --- | --- | --- |
| Increased temperature & reduced humidity | Yield (log) | -0.31 | 0.08 | 0.57 | <0.001 |
| Increased temperature & reduced humidity | Plant health (SPAD) | 4.14 | 0.60 | 0.51 | <0.001 |
| Increased temperature & reduced humidity | Powdery mildew cover per bunch (logit) | 1.47 | 0.44 | 0.51 | 0.003 |
| Increased temperature & reduced humidity | Botrytis cover per bunch (logit) | -2.66 | 0.23 | -0.92 | <0.001 |
| Number of ants (square root) | Number of mealybugs (log) | 0.19 | 0.06 | 0.52 | 0.002 |
| Increased temperature & reduced humidity | Number of mealybugs (log) | 0.49 | 0.73 | 0.12 | 0.512 |
| *~~Botrytis cover per bunch (logit)* | *~~Powdery mildew cover per bunch (logit)* | *-0.52* | *NA* | *-0.52* | *0.003* |

**Table J:** 2010/1 Field season path analysis output, with total yield as the main response variable of interest. Model 3 – as Model 2, but including additional hypothesized paths (in *italics*). SPAD = value from the Soil Plant Analysis Development meter. ~~ indicates that the pathway represents correlated errors. Other terms in parentheses refer to data transformation applied to the variable. Model statistics: Fishers C = 28.17, df = 20, p = 0.106, AICc = 755.61

| **Predictor variable** | **Response variable** | **Unstandardised path coefficient** | **Standard error** | **Standardised Path coefficient** | **p-value** |
| --- | --- | --- | --- | --- | --- |
| Increased temperature & reduced humidity | Yield (log) | -0.35 | 0.09 | 0.65 | 0.001 |
| Powdery mildew cover per bunch (logit) | Yield (log) | -0.03 | 0.03 | -0.15 | 0.395 |
| Increased temperature & reduced humidity | Plant health (SPAD) | 4.14 | 0.60 | 0.51 | <0.001 |
| Increased temperature & reduced humidity | Powdery mildew cover per bunch (logit) | 1.47 | 0.44 | 0.51 | 0.003 |
| Increased temperature & reduced humidity | Botrytis cover per bunch (logit) | -2.66 | 0.23 | -0.92 | <0.001 |
| Number of ants (square root) | Number of mealybugs (log) | 0.19 | 0.06 | 0.52 | 0.002 |
| Increased temperature & reduced humidity | Number of mealybugs (log) | 0.49 | 0.73 | 0.12 | 0.512 |
| ~~Botrytis cover per bunch (logit) | ~~Powdery mildew cover per bunch (logit) | -0.52 | NA | -0.52 | 0.003 |
| *Number of mealybugs (log)* | *~~Powdery mildew cover per bunch (logit)* | *0.07* | *NA* | *0.07* | *0.366* |
| *Number of mealybugs (log)* | *~~Botrytis cover per bunch (logit)* | *-0.06* | *NA* | *-0.06* | *0.386* |

**Table K:** 2010/11 Field season path analysis output, with total yield as the main response variable of interest. Model 4 – as model 1 in Table S8, but replacing the direct link between Yield and Temperature treatment with indirect links via powdery mildew and *Botrytis*. SPAD = value from the Soil Plant Analysis Development meter. Other terms in parentheses refer to data transformation applied to the variable. Model statistics: Fishers C = 107.95, df = 26, p = 0, AICc = 1526.80

| **Predictor variable** | **Response variable** | **Unstandardised path coefficient** | **Standard error** | **Standardised Path coefficient** | **p-value** |
| --- | --- | --- | --- | --- | --- |
| Powdery mildew cover per bunch (logit) | Yield (log) | -0.07 | 0.03 | -0.38 | 0.032 |
| Botrytis cover per bunch (logit) | Yield (log) | -0.16 | 0.03 | -0.87 | <0.001 |
| Increased temperature & reduced humidity | Plant health (SPAD) | 4.14 | 0.60 | 0.80 | <0.001 |
| Increased temperature & reduced humidity | Powdery mildew cover per bunch (logit) | 1.47 | 0.44 | 0.51 | 0.003 |
| Increased temperature & reduced humidity | Botrytis cover per bunch (logit) | -2.66 | 0.23 | -0.92 | <0.001 |
| Increased temperature & reduced humidity | Number of mealybugs (log) | 0.49 | 0.73 | 0.12 | 0.512 |
| Number of ants (square root) | Number of mealybugs (log) | 0.19 | 0.06 | 0.52 | 0.002 |

**Table J:** 2010/11 Field season path analysis output, with total yield as the main response variable of interest. Model 5 – as Model 4, but including the covariance path between Botrytis and Powdery Mildew (in *italics*). SPAD = value from the Soil Plant Analysis Development meter. ~~ indicates that the pathway represents correlated errors. Other terms in parentheses refer to data transformation applied to the variable. Model statistics: Fishers C = 29.05, df = 24, p = 0.218, AICc = 764.16

| **Predictor variable** | **Response variable** | **Unstandardised path coefficient** | **Standard error** | **Standardised Path coefficient** | **p-value** |
| --- | --- | --- | --- | --- | --- |
| Powdery mildew cover per bunch (logit) | Yield (log) | -0.07 | 0.03 | -0.38 | 0.032 |
| Botrytis cover per bunch (logit) | Yield (log) | -0.16 | 0.03 | -0.87 | <0.001 |
| Increased temperature & reduced humidity | Plant health (SPAD) | 4.14 | 0.60 | 0.80 | <0.001 |
| Increased temperature & reduced humidity | Powdery mildew cover per bunch (logit) | 1.47 | 0.44 | 0.51 | 0.003 |
| Increased temperature & reduced humidity | Botrytis cover per bunch (logit) | -2.66 | 0.23 | -0.92 | <0.001 |
| Increased temperature & reduced humidity | Number of mealybugs (log) | 0.49 | 0.73 | 0.12 | 0.512 |
| Number of ants (square root) | Number of mealybugs (log) | 0.19 | 0.06 | 0.52 | 0.002 |
| *~~Botrytis cover per bunch (logit)* | *~~Powdery mildew cover per bunch (logit)* | *-0.52* | *NA* | *-0.52* | *0.003* |

**Table K:** 2010/11 Field season path analysis output, with total yield as the main response variable of interest. Model 6 – as Model 5, but including additional hypothesized paths (in *italics*). SPAD = value from the Soil Plant Analysis Development meter. ~~ indicates that the pathway represents correlated errors. Other terms in parentheses refer to data transformation applied to the variable. Model statistics: Fishers C = 23.29, df = 20, p = 0.275, AICc = 708.48

| **Predictor variable** | **Response variable** | **Unstandardised path coefficient** | **Standard error** | **Standardised Path coefficient** | **p-value** |
| --- | --- | --- | --- | --- | --- |
| Powdery mildew cover per bunch (logit) | Yield (log) | -0.07 | 0.03 | -0.38 | 0.032 |
| Botrytis cover per bunch (logit) | Yield (log) | -0.16 | 0.03 | -0.87 | <0.001 |
| Increased temperature & reduced humidity | Plant health (SPAD) | 4.14 | 0.60 | 0.80 | <0.001 |
| Increased temperature & reduced humidity | Powdery mildew cover per bunch (logit) | 1.47 | 0.44 | 0.51 | 0.003 |
| Increased temperature & reduced humidity | Botrytis cover per bunch (logit) | -2.66 | 0.23 | -0.92 | <0.001 |
| Increased temperature & reduced humidity | Number of mealybugs (log) | 0.49 | 0.73 | 0.12 | 0.512 |
| Number of ants (square root) | Number of mealybugs (log) | 0.19 | 0.06 | 0.52 | 0.002 |
| *~~Botrytis cover per bunch (logit)* | *~~Powdery mildew cover per bunch (logit)* | *-0.52* | *NA* | *-0.52* | *0.003* |
| *Number of mealybugs (log)* | *~~Powdery mildew cover per bunch (logit)* | *0.07* | *NA* | *0.07* | *0.366* |
| *Number of mealybugs (log)* | *~~Botrytis cover per bunch (logit)* | *-0.06* | *NA* | *-0.06* | *0.386* |


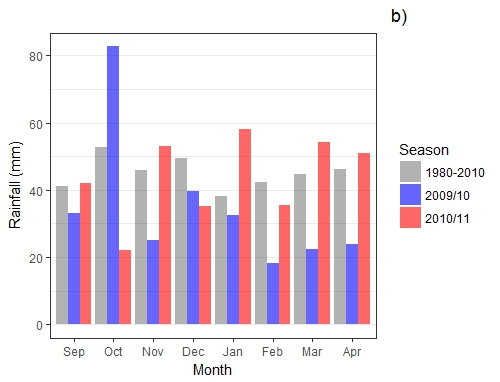

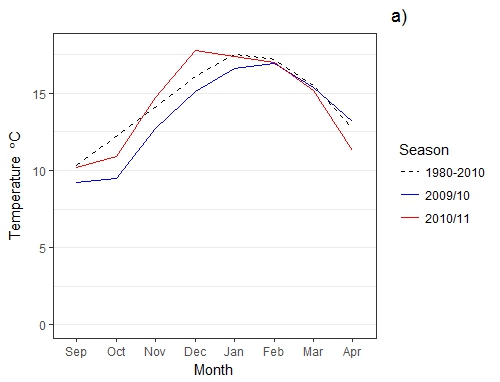


**Fig A.** a) Mean monthly temperature for Christchurch, New Zealand from 1980 to 2010 and during the two growing seasons. b) Monthly rainfall (mm) for Christchurch during the two growing seasons. All data recorded by The National Institute of Water and Atmospheric Research (NIWA), at the Christchurch Gardens station, New Zealand (43.533°S, 172.617°E, approx 20km from study site).
